# A Case Report of Traumatic Ulcerative Granuloma With Stromal Eosinophilia (TUGSE): Clinical and Histopathological Diagnostic Challenges

**DOI:** 10.7759/cureus.48481

**Published:** 2023-11-08

**Authors:** Ahmad Fakrurrozi Mohamad, Nawal Radhiah Abdul Rahman, Ewe Seng Ch'ng

**Affiliations:** 1 Department of Dental Science, Advanced Medical and Dental Institute, Universiti Sains Malaysia, Pulau Pinang, MYS; 2 Department of Clinical Medicine, Advanced Medical and Dental Institute, Universiti Sains Malaysia, Pulau Pinang, MYS

**Keywords:** cd30-positive lymphoproliferative disorders, langerhans cell histiocytosis, stromal eosinophilia, traumatic granuloma, tugse, eosinophilic ulcer, oral ulcer

## Abstract

Traumatic ulcerative granuloma with stromal eosinophilia is a reactive, self-limiting ulcer within the oral cavity; however, clinically, it mimics a malignant ulcer. Here, we report the case of a 13-year-old boy who presented with a painful solitary indurated ulcer at the lower lip for a week. Histopathological examination revealed an ulcerated lesion with significant infiltration of eosinophils, small lymphocytes, and large lymphoid cells. Further immunohistochemistry showed the inflammatory cells were CD3-positive T cells and CD68-positive, with a minority of the cell population showing CD30 positivity. CD1a-positive dendritic cells were also observed. We discuss the clinical and histopathological differential diagnoses of Langerhans cell histiocytosis and CD30-positive T-cell lymphoproliferative disorders and how to correlate them in formulating the final diagnosis.

## Introduction

Traumatic ulcerative granuloma with stromal eosinophilia (TUGSE) is a reactive, benign, self-limiting lesion highly associated with trauma [1-3]. Non-trauma-related TUGSE cases have also been reported, suggesting that factors other than trauma may contribute to its pathogenesis [1,3].

TUGSE manifests clinically as a rapidly developing solitary ulcer in oral soft tissue, especially on the dorsum and lateral border of the tongue [1,3,4]. Other common sites include buccal mucosa, followed by the retromolar area, floor of the mouth, lip, gingiva, and palate [1,3-5]. The ulcer may have a white or yellowish base surrounded by erythematous mucosa, which is indurated and has elevated borders [3,6,7]. Pain is commonly associated with the ulcer, with mild to severe pain [1,4,6]. TUGSE typically appears as a single lesion and seldom recurs [1,4]. The size of the ulcer varies from 0.5 cm to 6.5 cm [3]. Both genders are affected, with no consistent gender predilection reported [1,4,5,7]. TUGSE can also affect all ages, however, with two peak incidences. It can be observed in babies below two years old, mainly associated with teething, and in adults in their fourth to seventh decades of life [1,3,4,7]. Many TUGSE cases resolve without intervention, showing spontaneous healing, usually within a month, and sometimes may take up to a year [2,8]. However, as the clinical appearance of TUGSE mandates the exclusion of malignant diseases, an incisional biopsy of the lesion is usually performed. Most TUGSE cases heal rapidly after biopsy [2,8-10].

Histologically, granulation tissue underneath the ulcer comprises a dense, mixed cellular infiltrate with the predominant large mononuclear cells with pale nuclei harboring small nucleoli [3,4]. Eosinophils are abundant but may be sparse in some cases [1]. These cells are mainly found within the submucosa but are also seen penetrating between the muscle fibers and minor salivary glands [1,3,4]. The large mononuclear cells have been reported to represent predominantly CD30-positive T cells [4,5] and, to a lesser extent, CD3- and CD8-positive T cells [4]. TUGSE has been related to CD30-positive lymphoproliferative disorder [11].

This case report aims to highlight a case of lower lip TUGSE in a child and review the clinical and histopathological challenges in diagnosing the case.

## Case presentation

We detail the case of a 13-year-old male, a never-smoker, who was referred to the Oral and Maxillofacial Clinic at the Advanced Medical and Dental Institute, Universiti Sains Malaysia, for further evaluation of a solitary ulcer on the lower labial mucosa. The patient presented with a chief complaint of an ulcer on the lower lip for one week. He had mild pain and swelling on his lower lip, affecting his facial appearance. The patient denied experiencing any fever, night sweats, or weight loss. He was fit and well and not on any medications. In the past two months, he had a history of accidentally biting his lip in the same spot, which resulted in an ulcer that resolved on its own after a week. His past medical history was non-contributory.

**Figure 1:**
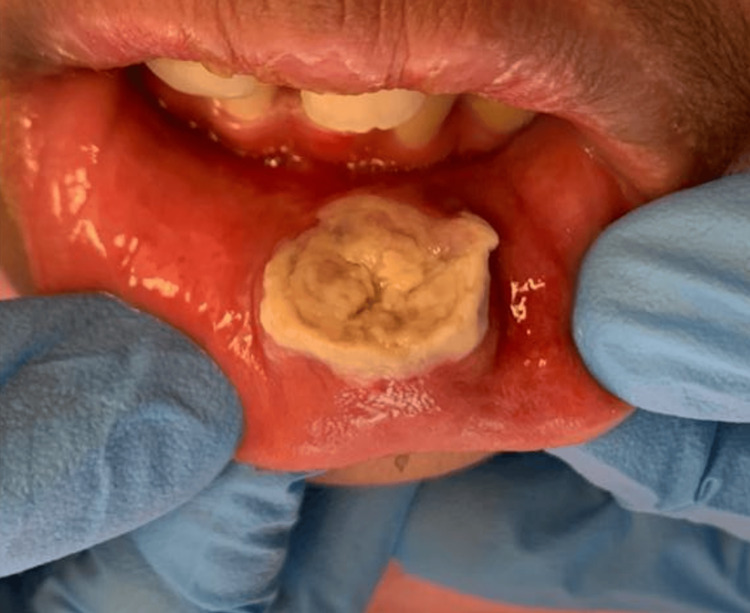
Lesion on the labial mucosa of the lower lip showing a large necrotic ulcer with raised, rolled margins.

Extraoral examination showed swelling and slightly everted lower lip without any skin redness. Palpation of the cervical lymph nodes revealed an enlarged submandibular lymph node at level Ib on the left side, which was mobile and had mild tenderness. Intraoral examination revealed a whitish, indurated ulcer with a rolled margin at the lower labial mucosa measuring 2 cm × 1.5 cm in dimension (Figure 1). It was firm in consistency and tender on palpation. Oral hygiene was fair, with generalized stains and calculus. He had a class II division 1 incisor relationship with a lower lip trap.

The rapid growth of the ulcer over the last seven days was potentially indicative of a malignant process. After obtaining informed consent from the parent, an incisional biopsy was performed on the margin of the ulcer under local anesthesia to obtain a histopathological diagnosis. Postoperatively, the patient was given analgesic medication when necessary, 0.2% chlorhexidine mouthwash to be used three times daily for one week, and topical gel consisting of hyaluronic acid for topical application at the lesion.

**Figure 2:**
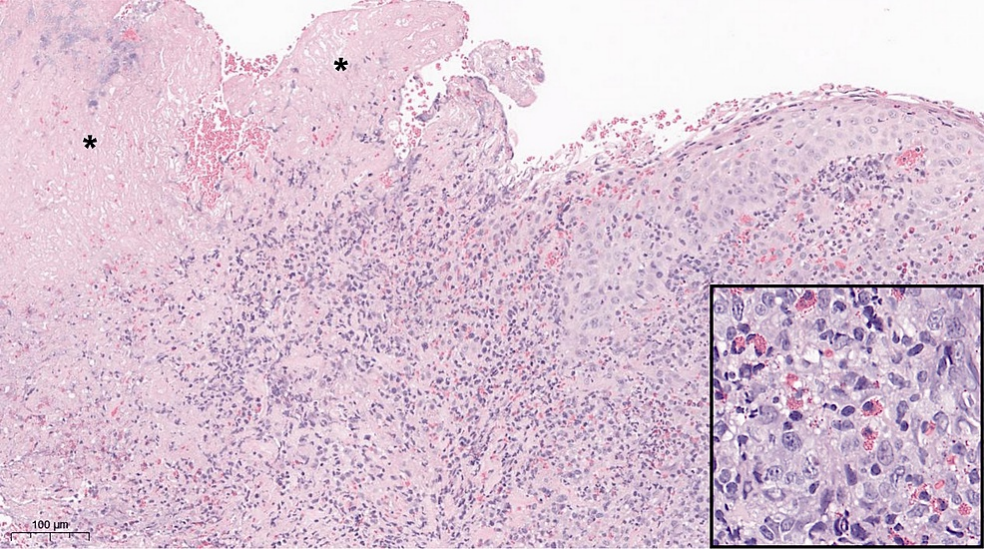
Low magnification of the photomicrograph of the specimen showing mucosal ulceration (*) and dense inflammation within the connective tissue. Inset photomicrograph demonstrates higher magnification of eosinophils seen along with lymphocytes and large mononuclear cells with vesicular nuclei harboring small nucleoli and a rim of amphophilic cytoplasm.

Histologically, the mucosa fragments were ulcerated with fibrinopurulent exudate rich in eosinophils. There were dense heterogeneous inflammatory infiltrates in the stroma composed of eosinophils, small lymphocytes, large mononuclear cells with round to oval pale nuclei, and histiocytes. No increased mitosis or abnormal mitotic figures were seen (Figure 2).

**Figure 3:**
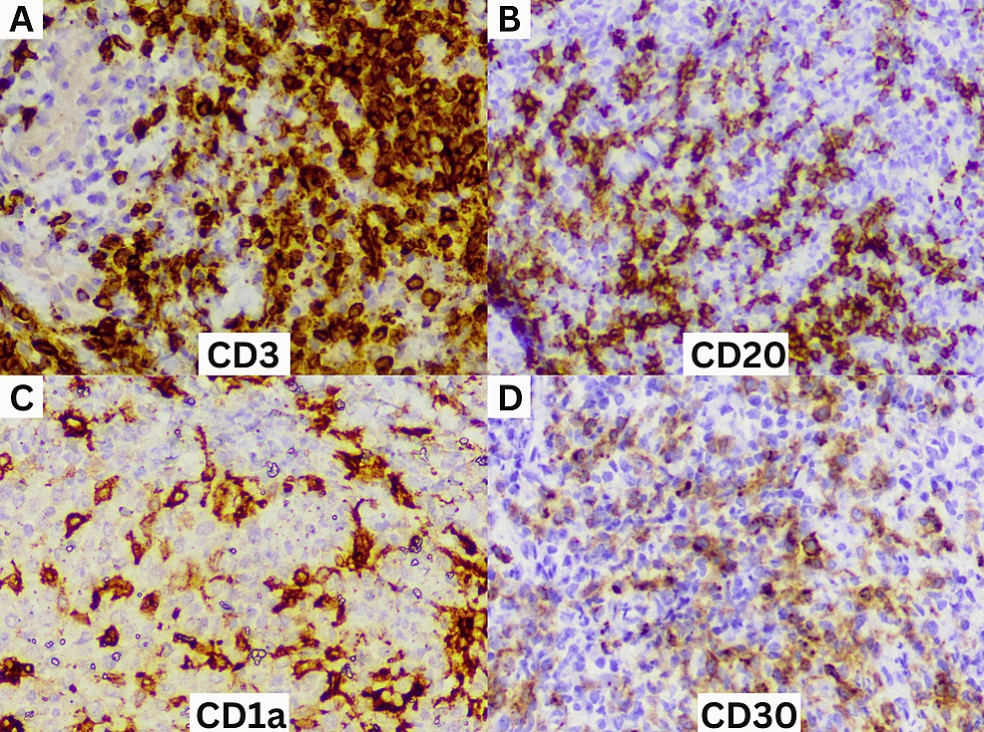
Immunohistochemistry of the specimen marking the cell population comprising mainly CD3-positive T cells (A), some CD20-positive B cells (B), CD1a-positive dendritic cells (C), and CD30-positive large mononuclear cells (D). Original magnification 20×.

Immunohistochemistry showed that the inflammatory infiltrates comprised predominantly CD3-positive T cells (Figure 3A), CD20-positive B cells were seen in a minor population (Figure 3B), and CD68-positive cells were evenly distributed. CD1a immunohistochemistry highlighted scattered CD1a-positive dendritic cells among the inflammatory infiltrates but not the large mononuclear cells (Figure 3C). A proportion of large mononuclear cells were CD30 positive (Figure 3D).

**Figure 4:**
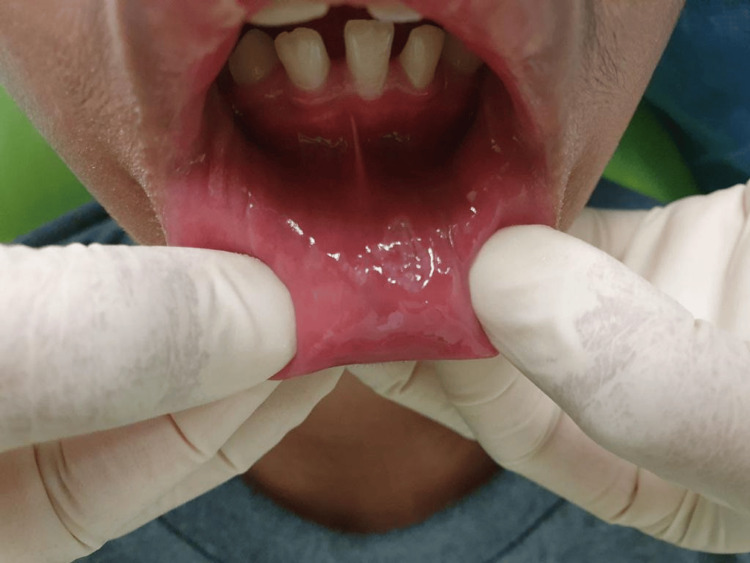
The lesion appearance two weeks after the incisional biopsy showing uneventful healing.

The patient was recalled after two weeks; the lesion showed significant regression, and the patient was pain-free (Figure 4). An oral soft splint was fabricated to cover the protruding upper teeth to prevent future trauma. A dental panoramic tomograph was obtained to rule out any bony lesion, and no bony lesion was detected. He was then referred to an orthodontist for the management of dental malocclusion.

Based on the combined clinical and histopathological features, a definitive diagnosis of TUGSE was established. Eighteen months post-diagnosis, no ulceration recurrence or scarring was observed on the lower labial mucosa.

## Discussion

TUGSE is a rare and intriguing condition in pathology, previously known as Riga-Fede’s disease or eosinophilic ulcer. It was first described in the late 19th century by Italian physician Antonio Riga and later by Romanian pathologist Angelo Fede, thus termed Riga-Fede’s disease [7]. Riga-Fede’s disease was primarily described as newborns’ lingual frenums becoming ulcerated due to mechanical damage caused by nursing on erupting teeth [7]. Elzay suggests that TUGSE includes Riga-Fede’s disease and eosinophilic ulcer, regardless of the patient’s age. Many authors believe the lesions are traumatic in origin and are brought on by accidental bites or repeated thrusting against sharp, misplaced, or fractured teeth [1,6]. According to Vélez et al., trauma is only a contributing factor in the development of TUGSE, which may be caused by viral or toxic agents penetrating the underlying tissue and triggering an inflammatory response [10]. This hypothesis is supported by the increased incidence of this lesion on the tongue and buccal mucosa, which are easily exposed to trauma during chewing, and the definitive history of traumatic injury in one-third to one-half of reported cases [10,12,13].

The differential diagnosis of ulcerative tongue lesions should include traumatic ulcers and infectious diseases such as primary syphilis, tuberculosis, or Epstein-Barr virus-associated mucocutaneous ulcers. Lymphoma, metastasis disease, reactionary proliferative processes such as atypical histiocytic granuloma, proliferative myositis, and squamous cell carcinoma may present with the same clinical features and should be included in the differential diagnosis.

Histologically, the inflammatory infiltrate comprised eosinophils, small lymphocytes, and large lymphoid cells. A significant number of eosinophils are seen in TUGSE compared to non-specific ulcers [14], rendering eosinophils pathognomonic in TUGSE. However, the number of eosinophils can range from mild to moderate [1] or even sparse [15]. These eosinophils can be seen within the granulation tissue, and some may extend into the superficial and deep muscle layer [1]. The delayed healing of TUGSE has been suggested due to reduced expression of transforming growth factor alpha and transforming growth factor beta 1 of the eosinophils compared to normal wound healing [2]. In addition, the degranulating eosinophils and the cytotoxic activity of the T cells are associated with the mucosal degeneration of TUGSE lesions [2,3]. The evidence of cytotoxic activity of the T cells was supported by the abundance of T-cell intracytoplasmic antigen 1, a cytotoxic T cell component in a TUGSE case series [16].

On the other hand, the presence of eosinophils within the heterogeneous inflammatory cells resembles Langerhans cell histiocytosis (LCH). LCH is a rare disorder involving abnormal proliferation of histiocytes [17]. Eosinophils are commonly observed within LCH; however, the number of eosinophils may vary from abundant to sparse or absent [18]. As the name implies, the pathognomonic cells for LCH are the Langerhans cell histiocytes, the immature dendritic cells without cell extensions [17]. The cells have amphophilic to eosinophilic cytoplasm, with characteristic “coffee bean”-shaped or folded or indented oval nuclei or grooved nuclei with small or absent nucleoli [18]. They are positive for CD1a, CD207/langerin, and S100 protein [17]. However, CD1a may occasionally be positive in other histiocytic proliferations as CD1a also identifies cortical thymocytes and interdigitating dendritic cells [17]. Thus, langerin or CD-207, a cell surface protein that leads to Birbeck granule formation in Langerhans cells, is more specific and sensitive in identifying Langerhans cells [19]. Birbeck granules are rod-shaped structures within the cytoplasm of the dendritic cells, which can only be seen under electron transmission microscopy [17,19].

Nonetheless, clinically, LCH in the oral and maxillofacial region usually involves the jawbone, especially the posterior mandible. A dental panoramic tomogram usually shows ill-defined radiolucency that can be unilocular or multilocular. Bony lesions may be accompanied by soft tissue swelling or ulcers. In this case, a minority of dendritic cells expressed CD1a. However, due to the clinical site (lower lip), this was unlikely to represent LCH.

In this case, a portion of large lymphoid cells was positive for CD30. CD30 is a lymphocyte activation antigen and a member of the tumor necrosis factor family [15]. Its presence is diagnostic of primary cutaneous CD30-positive T-cell lymphoproliferative disorders, classic Hodgkin’s lymphoma, anaplastic large-cell lymphoma, and embryonal carcinoma [15]. However, this is not an uncommon presentation in TUGSE. Studies concerning CD30 show that 70% of TUGSE cases have been expressed within the large, atypical cells, either strong or focal positivity, with CD30 [5], and 40% of cases have scattered positivity [16]. However, non-specific staining involving both large, atypical cells and small lymphoid cells was also observed in another study [4].

This led to the debate about whether this expression may or may not be associated with primary CD30-positive T-cell lymphoproliferative disorder (CD30-positive TLPD). Some authors did suggest TUGSE might be an oral counterpart of primary CD30-positive TLPD [11,13]. However, Aizic et al. found TUGSE to be reactive rather than neoplastic, even in cases with CD30-positive atypical cells [14]. In addition, Argyris et al. studied a case series of CD30-positive TLPD of the gingiva. They compared it with TUGSE, suggesting that parameters such as a diffuse sheet-like growth pattern of CD30-positive cells, the affinity of lesional cells toward neurovascular elements (angiocentric and neurotropism), the presence of Hallmark cell (eccentric, kidney-shaped nuclei with abundant cytoplasm), and atypical mitotic figures would favor diagnosis toward CD30-positive TLPD [20]. Interestingly, CD30-positive TLPD of the gingiva in the case series also self-regressed without adjuvant therapy [20]. In our case, the parameters mentioned earlier were not present.

Management and treatment

Our case demonstrated a rapid improvement of the ulcer following the incisional biopsy, which aligns with the experiences of other authors. This highlights the effectiveness of the procedure and its potential to lead to positive outcomes. Surgical intervention may favor the reactivation of the healing response, but the reason for this improvement remains unknown [9].

Surgical excision is the most commonly cited treatment [12]. Despite no evidence of efficacy, topical steroids or mouthwashes can be prescribed. Other therapeutic modalities include intralesional steroids, topical antibiotics, curettage, and cryotherapy [8]. No further local recurrences are usually noted after excision. However, recurrence can occur if the underlying cause of trauma is not addressed [1]. It is possible to develop new lesions in other mucosal areas [6]. Thus, the mainstay of treatment is to eliminate any apparent source [9]. As in our case, the patient was prescribed an oral soft splint to prevent further trauma from his upper anterior teeth and then referred for orthodontic management of his malocclusion.

Although TUGSE is a benign affection, monitoring is mandatory in all cases, as low-grade lymphoma such as primary CD30-positive TLPD may show a similar clinical presentation in the oral mucosa. Our case did not exhibit a recurrence after six months, as is typically reported in TUGSE [1,4,16].

## Conclusions

TUGSE is a rare ulcerative oral mucosal lesion. Because TUGSE frequently mimics infectious diseases or malignancies, a biopsy and a thorough clinical examination are required for diagnosis. In most instances, the lesions tend to heal on their own after the surgical intervention, rendering any further treatment unnecessary. Nevertheless, it is still advisable to undergo clinical monitoring. The duration of clinical monitoring for a patient is not specified by any fixed protocol. Instead, the expert advice of clinicians is recommended to determine the appropriate management approach.
